# Balancing grain and forage production in dual-purpose cereals: physiological basis, yield variation, and economic evaluation

**DOI:** 10.1186/s12870-026-08446-5

**Published:** 2026-02-26

**Authors:** Dan Wu, Yuanyan Meng, Xianfu Lv, Qingfu Huang, Mingyue Haung, Hua Jiang, Liuxing Xu

**Affiliations:** 1 College of Agronomy and Life Sciences, Zhaotong University, Zhaotong, 657000 China; 2 School of Geographical Sciences and Tourism, Zhaotong University, Zhaotong, Yunnan 657000 China; 3 Faculty of Animal Science and Technology, Yunnan Agricultural University, Kunming, 650201 China

**Keywords:** Dual-purpose crop, Economic benefits, Grain, Yield

## Abstract

Dual-purpose crops have become an indispensable part of crop-livestock systems. However, selecting suitable crops and implementing dual-purpose management pose unpredictable challenges to achieving suitable yields and economic returns. This study analyzed the effects of two treatments (grain and grain + forage) on the yield and photosynthetic and antioxidant properties of four crops (barley, triticale, oat, and wheat) over three growing seasons (2021–2022, season 1; 2022–2023, season 2; and 2023–2024, season 3) and assessed the yield and economic benefits. Compared to oat, barley, and triticale, grain + forage reduced (*P* < 0.05) the fresh matter and dry matter yields of wheat in the post-cutting growth period and grain yield at harvest. The fresh matter and dry matter yields at harvest of grain + forage were reduced (*P* < 0.05) by 31.7% and 21.5% compared with those of grain only, respectively. The factor analysis model constructed based on photosynthetic and antioxidant indicators (13 indicators) was ordered season 1 > season 2 > season 3 based on season, oat > wheat > triticale > barley based on crop, and grain only > grain + forage based on cropping system. Dual-purpose cropping demonstrated economic viability and sustainability by improving the harvest index despite reduced grain yield. However, the instability of rainfall has become the most critical natural factor limiting the promotion of dual-purpose crop management. When considering dual-purpose or grain-only cropping systems, oat emerged as the optimal choice, whereas for forage production, barley was the ideal singular crop.

**Supplementary Information:**

The online version contains supplementary material available at 10.1186/s12870-026-08446-5.

## Introduction

Dual-purpose crop systems allow cereals to be grazed or cut during the vegetative growth stage to provide forage, while still allowing for grain harvest at maturity [1]. These crops serve as valuable forage resources in countries and regions, such as Australia [2], the United States [3], Brazil [4], the Mediterranean [5], and the Loess Plateau [6]. Dual-purpose crops can provide ruminants with high-nutrient forage (2.3–2.4 t hm^− 2^ of biomass yield) and fill the seasonal feed gap [7]. However, this management strategy carries risks; insufficient fertilizer input [6] or harsh climatic conditions, such as drought or hail, can lead to grain yield declines [6, 8]. Nevertheless, the system remains profitable as long as the income from forage compensates for any potential loss in grain yield [9, 10]. Beyond grazing, whole-plant cereal crops (harvested for silage) are also significant. Their dry matter yield can reach 10.6–16.0 t ha^− 1^ dry matter, with starch concentrations ranging from 5.8% to 19.0% of dry matter [11]. These crops possess high nutritional value and excellent silage fermentation quality, making them an essential source of roughage in animal husbandry [12]. In some scenarios, such as when rainfall changes [13] or overgrazing leads to lower grain yields [14], utilizing whole-plant cereal crops in their entirety becomes economically viable. Consequently, dual-purpose crop systems may incorporate novel approaches, including grazing at the vegetative growth phase and whole-plant at the reproductive growth phase (milk or dough stage).

Despite these options, the specific effects of early grazing (cutting) on the final whole-plant yield and physiological traits at maturity remain poorly understood. Furthermore, unpredictable yield outcomes and economic returns make it challenging to select appropriate crops and management strategies [2, 15]. While previous research has explored various crop types and practices, results often vary due to geographic and climatic differences. Therefore, integrating findings from regional trials is crucial for optimizing the balance between grain and forage production.

This study conducted a three-year continuous field experiment involving four crop varieties (*Hordeum vulgare* L., *Avena sativa* L., *Triticale wittmack* L., and *Triticum aestivum* L.) and two management methods. We aimed to systematically explore how dual-purpose cropping strategies affect photosynthetic and antioxidant properties, and how these physiological changes correlate with grain/forage yields and economic benefits. By comparing these results with practical cases from diverse climatic regions (arid vs. high-rainfall), we aimed to establish a comprehensive management strategy. The research hypotheses were: (1) wheat exhibits superior antioxidant activities compared to barley, oat, and triticale; (2) cutting increases grain yield potential but may reduce whole-plant yield and harvest index.

## Materials and methods

### Experimental site

**Fig. 1:**
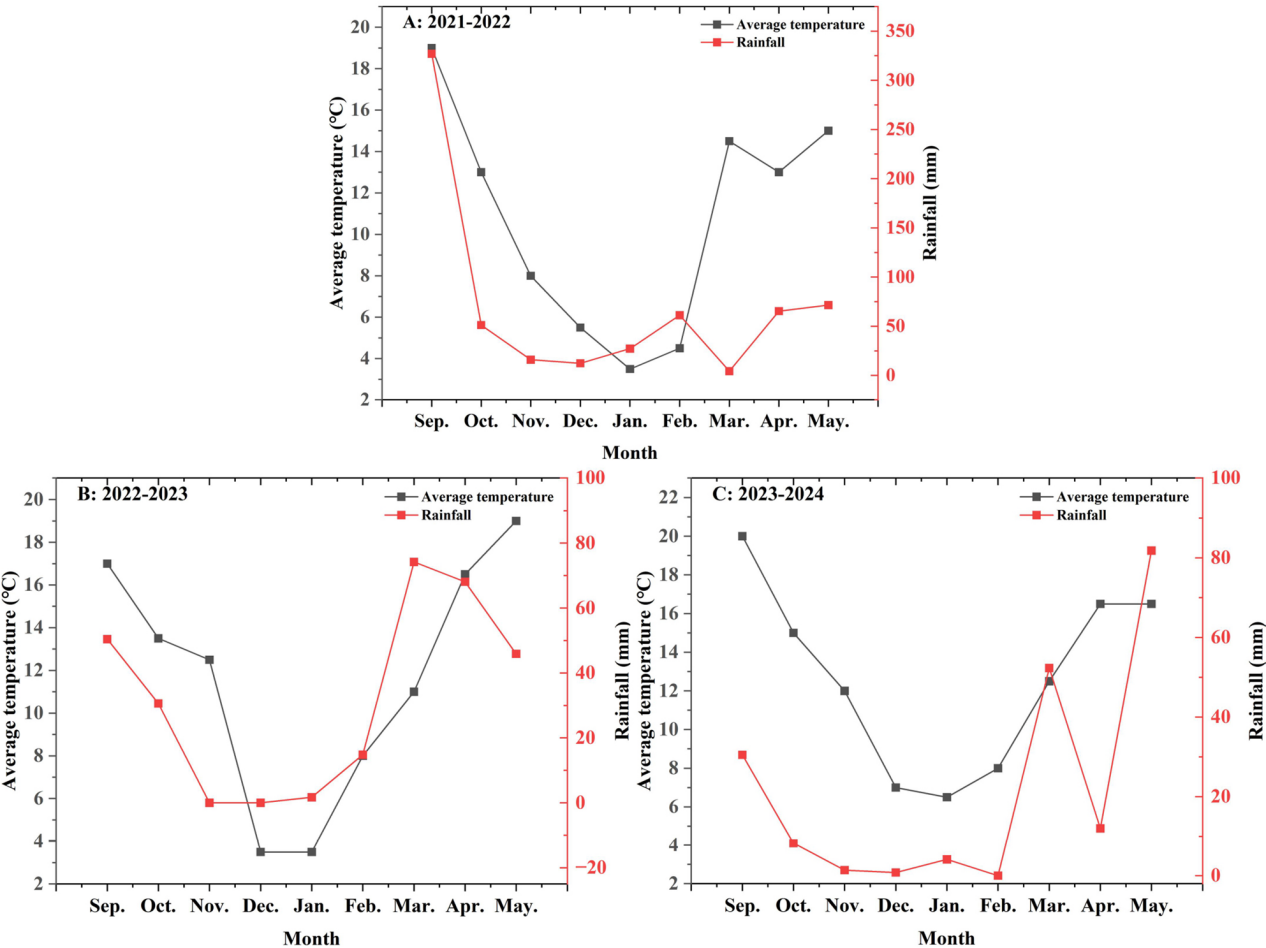
Meteorological data (temperature and rainfall) during the growing periods of crops across three seasons (2021–2024)

The experimental site was set up at the experimental field of Zhaotong University (Zhaoyang District, Zhaotong City, Yunnan Province; 27º36′N, 103º74′E; 1985 m altitude). According to data from the Zhaotong Meteorological Bureau, the average annual temperature over the past 20 years was 12.3 °C and with an average annual rainfall of 682 mm. The total rainfall and average temperature during the crop growth periods of 2021–2022 (season 1), 2022–2023 (season 2), and 2023–2024 (season 3) were 636 mm and 10.7 °C, 286 mm and 11.6 °C, and 191 mm and 12.7 °C, respectively. Overall, the temperature during the crop growth period was relatively low (Fig. 1). After soybean [*Glycine max* (L.) Merr] harvest (Sep.), dual-purpose crops were cultivated (harvested at the end of the following spring), followed by soybean cultivation again. Soil data were not collected at the time of the site establishment.

### Experimental treatment and crop management

Field experiments were conducted from Sep. 2021 to May. 2022 (season 1), Sep. 2022 to May. 2023 (season 2), and Sep. 2023 to May. 2024 (season 3). The trials included four crops: barley, triticale, oat, and wheat. Each crop was subjected to either grain-only or grain + forage treatments. The seeding rate was set at 3 × 10^6^ plant hm^− 2^. Manual tillage was employed, with seeds broadcast onto the soil surface and subsequently covered with 5–10 mm of soil. Each plot consisted of three replicates (3 m × 4 m) arranged randomly. The four crops were sown in Sep. and harvested in May (Table 1). A 40% compound fertilizer (N: P_2_O_5_: K_2_O = 2:1:1) was applied after emergence (Nov.). To explicitly support crop recovery and regrowth, the remaining 60% was applied as top-dressing at the jointing stage, immediately following the cutting treatment. Crucially, given the limited rainfall, supplemental irrigation (40 mm) was applied immediately after this top-dressing. This integrated post-cutting management (fertilization coupled with irrigation) aimed to facilitate rapid fertilizer dissolution and root uptake, thereby mitigating the impact of seasonal drought on crop regrowth across the different years. The growth stages of the crops were determined using a decimal code system [16]. Table 1 shows the detailed field management practices, stages, and growing times of the four crops in the different seasons. The difference in crop growth time among the three growing seasons reached up to 43 d. Four crops were cut at the jointing stage (based on cut identification), resulting in significant differences between the subsequent grain-only and grain + forage treatments at the vegetative growth stage due to the effects of cutting. No insecticides or fungicides were used during the experimental period.

**Table 1:** Growing time, stage, agronomic management of three crops

Year	Crop	Cut	Jointing stage (cut)		Flowering stage (cut)		Dough stage (cut)		Cutting time		First fertilizer date	Second fertilizer date	Sowing date	Harvest date	Total growth time (d)	
			Grain only	Grain + forage	Grain only	Grain + forage	Grain only	Grain + forage	Time	Crop growth time (d)					Grain only	Grain + forage
Season 1	Wheat	DC26	DC40	DC29	DC58	DC51	DC85	DC83	9 Nov.	61	10 Nov.	3 Jan.	10 Sep.	14 May.	247	186
	Oat	DC26	DC41	DC31	DC55	DC50	DC77	DC73								
	Barley	DC25	DC45	DC33	DC62	DC54	DC90	DC87								
	Triticale	DC28	DC47	DC34	DC66	DC56	DC92	DC88								
Season 2	Wheat	DC29	DC38	DC28	DC55	DC47	DC88	DC85	25 Nov.	60	17 Nov.	12 Dec.	27 Sep.	19 May.	204	144
	Oat	DC28	DC40	DC28	DC53	DC45	DC81	DC80								
	Barley	DC26	DC43	DC27	DC58	DC49	DC89	DC88								
	Triticale	DC28	DC45	DC29	DC60	DC52	DC90	DC89								
Season 3	Wheat	DC25	DC40	DC27	DC60	DC50	DC87	DC83	14 Dec.	66	20 Nov.	5 Jan.	10 Oct.	20 May.	224	158
	Oat	DC26	DC39	DC27	DC50	DC43	DC82	DC81								
	Barley	DC24	DC43	DC32	DC50	DC47	DC89	DC87								
	Triticale	DC26	DC47	DC33	DC56	DC50	DC82	DC87								

*DC* Decimal code

### Field sampling

At the jointing stage, a quadrat of 1 m^2^ (1 m × 1 m) was selected from each plot (cut 2 cm above the ground) to measure the fresh matter yield. After the fresh matter yield was measured, 200 g of fresh material was selected and taken back to the laboratory for drying (at 70 °C for 48 h) to determine the dry matter content. The dry matter yield was then calculated using the dry matter content and fresh matter yield. The whole-plant yield at the dough stage was measured using a similar method. A new quadrat of 1 m^2^ (1 m × 1 m) was selected to determine the grain yield and kernel weight. At the milk and dough stages, the flag leaf was selected to measure photosynthetic and antioxidant properties. The agronomic traits and photosynthetic properties were measured directly in the field.

### Photosynthetic and antioxidant property analyses

Photosynthetic properties: On a sunny morning, an LI-6800 photosynthesis system (LI-COR, Lincoln, NE, USA) was used to measure the net photosynthetic rate (Pn), intercellular carbon dioxide concentration (Ci), transpiration rate (Tr), and pore conductivity of the water vapor (Gs) of the crops (selecting the middle part of the leaves while avoiding the veins). Following these measurements, we calculated the water use efficiency (WUE, in units of µmol mmol^− 1^) using the formula WUE = Pn/Tr. The parameters of the photosynthesis system were set as follows: flow rate. 500 µmol s^− 1^; relative humidity, 50%; CO_2_ concentration, 400 µmol mol^− 1^; fan speed, 10,000 rpm; and light intensity, 1500 µmol m^− 2^ s^− 1^.

Antioxidant properties: The anthrone colorimetric method was used to determine soluble sugar content [17]. Ninhydrin was employed as a developer to measure the free amino acid content [18]. The total phenolic content was determined by reducing tungstomolybdic acid to blue using polyphenols [19]. After homogenization with 50 mM phosphate buffer (pH 7.8), the malondialdehyde content was determined [20]. To measure the ascorbate peroxidase activity, a reaction mixture containing 50 mM phosphate buffer (pH 8.0), 0.1 mM EDTA, 0.5 mM ascorbate, and 0.1 mM H_2_O_2_ was used [21]. Peroxidase activity was assayed based on the degree of guaiacol oxidation at 470 nm according to a previously established protocol [22]. Catalase activity was determined using 100 mM phosphate buffer (pH 7.0), 0.1 mM EDTA, 0.1% H_2_O_2_, and 0.1 mL of enzyme extract (3 mL) as the reaction mixture [23]. Polyphenol oxidase activity was detected using catechol as the substrate [24].

### Statistical analysis

In this study, ANOVA was performed to test for normality and homogeneity of variance to evaluate the effects and interactions of seasons, crops, and cuttings on the agronomic traits, yield, photosynthesis, and antioxidant properties of dual-purpose crops (IBM SPSS Statistics 26, IBM Corp., Armonk, NY, USA; Duncan’s test). The model was constructed using the overall mean, treatment, and residuals [25]. Pearson’s correlation analysis was performed to determine the relationships among various factors. To gain a deeper understanding of the interrelationships between various indicators, principal component analysis (PCA) was conducted on two separate datasets: (1) crops and (2) dual-purpose (OriginPro^®^2022b, OriginLab Corp., Northampton, MA, USA).

## Results

### Variation in crop yield of pre-cutting and post-cutting growth rates of crops

Statistical analysis revealed that the season × crop interaction significantly influenced fresh (*P* < 0.001) and dry (*P* < 0.001) matter yields during the pre-cutting stage. During pre-cutting, a small difference was observed in the fresh and dry matter yields between seasons 1 and 2. Compared to oat, the fresh and dry matter yields of wheat, barley, and triticale increased (*P* > 0.05) by 0.63–0.87 t hm^− 2^ and 0.15–0.25 t hm^− 2^, respectively. During post-cutting, the fresh matter and dry matter yields in season 1 increased (*P* > 0.05) by 0.35 and 0.29 t hm^− 2^ relative to those in season 2, respectively. Notably, wheat exhibited the lowest post-cutting growth rate among the four crops. In contrast, oat, barley, and triticale showed significantly higher recovery, with fresh and dry matter yields increasing by 2.55–3.80 t hm^− 2^ and 1.25–1.58 t hm^− 2^, respectively, compared to wheat (*P* < 0.05) (Table 2).

**Table 2:** Growth rate of different seasons and crops (*n* = 24)

Seasons and crop types		Pre-cutting growth rate			Post-cutting growth rate	
		Dry matter content (g kg^− 1^, FM)	Fresh matter yield (t hm^− 2^)	Dry matter yield (t hm^− 2^)	Fresh matter yield (t hm^− 2^)	Dry matter yield (t hm^− 2^)
Seasons (S)	Season 1	162 ± 6.96b	2.66 ± 0.41	0.46 ± 0.08	5.95 ± 0.92	3.00 ± 0.32
	Season 2	182 ± 6.00a	2.62 ± 0.29	0.47 ± 0.05	5.60 ± 0.52	2.71 ± 0.23
Crops (C)	Wheat	185 ± 12.1a	2.83 ± 0.25	0.53 ± 0.07	3.22 ± 0.40b	1.82 ± 0.23b
	Oat	149 ± 9.08b	2.08 ± 0.52	0.31 ± 0.08	7.02 ± 0.79a	3.07 ± 0.20a
	Barley	168 ± 4.89ab	2.71 ± 0.28	0.46 ± 0.05	6.99 ± 1.16a	3.15 ± 0.44a
	Triticale	188 ± 2.58a	2.95 ± 0.78	0.56 ± 0.15	5.88 ± 0.95a	3.40 ± 0.31a
P value	S	0.040	0.933	0.863	0.744	0.473
	C	0.010	0.628	0.311	0.019	0.008
	S × C	0.021	< 0.0001	< 0.0001	0.070	0.644

Different lowercase letters in the same column represent significant difference between experiment seasons or crops (*P* < 0.05)

### Season and crop affected the kernel weight, grain yield, and fresh matter yield

Kernel weight and grain yield were determined using two sources of variation: season and crop (*P* < 0.05). Fresh matter yield was determined using three sources of variation: season, dual-purpose, and season × dual-purpose interactions (*P* < 0.05). Dry matter yield was determined using two sources of variation: crop and dual-purpose (*P* < 0.05). The harvest index was determined based on one source of variation: crop (*P* < 0.05). Compared to season 1, the kernel weight, grain, and fresh matter yields in season 2 decreased (*P* < 0.05) by 16.9%, 25.3%, and 16.5%, respectively. Although the fresh matter yield did not differ (*P* > 0.05) among the four crops, wheat had the lowest (*P* < 0.05) grain and dry matter yields. The harvest index of triticale and oat were significantly higher (*P* < 0.05) than those of barley and wheat. The fresh matter and dry matter yields of grain + forage were reduced (*P* < 0.05) by 31.7% and 21.5%, respectively, compared with those of grain only (Table 3).

**Table 3:** Agronomic traits and yield of different seasons, crops and dual-purpose (*n* = 66)

Seasons and treatments		Plant height (cm)	Stem number per plant	Kernel weight (g)	Grain yield (t hm^− 2^)	Fresh matter yield (t hm^− 2^)	Dry matter content (g kg^− 1^, FM)	Dry matter yield (t hm^− 2^)	Harvest index
Seasons (S)	Season 1	59.2 ± 2.54	11.8 ± 1.84a	32.6 ± 1.06a	3.12 ± 0.29a	7.99 ± 0.56b	38.6 ± 0.89	3.08 ± 0.23	0.40 ± 0.03
	Season 2	51.8 ± 2.76	5.63 ± 0.31b	27.1 ± 1.17b	2.33 ± 0.20b	6.67 ± 0.47b	38.6 ± 0.84	2.56 ± 0.18	0.36 ± 0.02
	Season 3	61.9 ± 4.78	2.98 ± 0.43b	/	/	13.8 ± 1.96a	/	/	/
Crops (C)	Wheat	60.0 ± 3.11ab	5.84 ± 1.08	33.2 ± 2.56a	1.36 ± 0.09d	7.31 ± 1.84	38.3 ± 1.24	1.61 ± 0.12b	0.32 ± 0.04b
	Oat	40.1 ± 2.59	6.67 ± 0.99	32.0 ± 1.11b	4.17 ± 0.22a	8.37 ± 0.40	35.6 ± 0.68	3.00 ± 0.18a	0.50 ± 0.04a
	Barley	55.0 ± 2.15b	10.7 ± 2.44	29.5 ± 0.99b	2.26 ± 0.22c	11.3 ± 1.06	36.7 ± 0.56	3.53 ± 0.24a	0.24 ± 0.02c
	Triticale	68.5 ± 3.92a	5.31 ± 0.91	24.7 ± 0.73b	3.10 ± 0.18b	9.12 ± 1.25	43.8 ± 0.56	3.15 ± 0.30a	0.45 ± 0.03a
Dual-purpose (DP)	Grain only	57.0 ± 2.31	6.65 ± 0.75	31.4 ± 1.07	2.99 ± 0.26	10.8 ± 1.15a	39.5 ± 0.84	3.16 ± 0.23a	0.38 ± 0.03
	Grain + forage	57.5 ± 3.12	7.67 ± 1.47	28.3 ± 1.34	2.46 ± 0.26	7.38 ± 0.65b	37.8 ± 0.85	2.48 ± 0.17b	0.38 ± 0.03
P value	S	0.085	< 0.0001	0.001	0.033	< 0.0001	0.981	0.079	0.295
	C	< 0.0001	< 0.0001	< 0.0001	< 0.0001	0.186	< 0.0001	< 0.0001	< 0.0001
	DP	0.906	0.540	0.079	0.151	0.012	0.170	0.021	0.983
	S × C	0.003	0.126	0.664	0.248	0.885	0.302	0.938	0.405
	S × DP	0.064	0.251	0.092	0.390	0.007	0.574	0.521	0.901
	C × DP	0.165	0.010	0.500	0.570	0.998	0.575	0.964	0.876

Different lowercase letters in the same column represent significant difference between experiment seasons, crops, or dual-purpose (*P* < 0.05)

### Influence of season and crop on photosynthetic properties

From seasons 1 to 3, the photosynthetic properties of the crops (excluding Ci) decreased (*P* < 0.05). Compared with at the milk stage, photosynthetic properties tended to decrease at the dough stage. Among the four crops, barley had the lowest photosynthetic properties, with its Tr and Gs significantly lower (*P* < 0.01) than those of oat and triticale. The Tr, Pn, Ci, and Gs of grain + forage were reduced (*P* < 0.05) by 40.1%, 29.2%, 24.4%, and 42.6%, respectively, compared to that of grain only, whereas the WUE increased (*P* < 0.05) by 20.0% (Table 4).

**Table 4:** Photosynthetic properties of different seasons, crops, dual-purpose, and maturity stages (*n* = 114)

Seasons and treatments		Tr (mmol H_2_O m^− 2^ s^− 1^)	Pn (µmol CO_2_ m^− 2^ s^− 1^)	Ci (µmol CO_2_ m^− 2^ s^− 1^)	Gs (mmol H_2_O m^− 2^ s^− 1^)	WUE (µmol mmol^− 1^)
Seasons (S)	Season 1	3.91 ± 0.32a	28.9 ± 2.09a	332 ± 17.4a	184 ± 16.7a	8.92 ± 0.72a
	Season 2	2.09 ± 0.16b	10.1 ± 0.56b	199 ± 11.2b	103 ± 8.53b	5.67 ± 0.38b
	Season 3	1.65 ± 0.16b	5.21 ± 0.87b	295 ± 8.22a	83.9 ± 9.33b	2.84 ± 0.26c
Maturity stages (MS)	Milk stage	2.89 ± 0.21	19.0 ± 2.07	288 ± 10.3a	147 ± 11.9a	6.10 ± 0.49
	Dough stage	2.66 ± 0.31	14.8 ± 1.26	245 ± 20.1b	117 ± 13.4b	7.26 ± 0.66
Crops (C)	Wheat	2.71 ± 0.36ab	14.6 ± 2.49	300 ± 27.3	138 ± 21.8ab	5.82 ± 0.61
	Oat	3.96 ± 0.47a	21.2 ± 2.65	269 ± 21.1	164 ± 18.9a	6.38 ± 0.65
	Barley	1.93 ± 0.17b	13.7 ± 1.88	227 ± 14.2	92.9 ± 8.72b	7.57 ± 0.90
	Triticale	3.01 ± 0.35a	20.3 ± 3.17	285 ± 17.4	147 ± 17.8a	6.55 ± 0.94
Dual-purpose (DP)	Grain only	3.50 ± 0.28a	20.2 ± 2.15a	308 ± 13.4a	170 ± 14.7a	5.56 ± 0.38b
	Grain + forage	2.08 ± 0.17b	14.3 ± 1.44b	233 ± 14.7b	97.5 ± 7.72b	7.62 ± 0.68a
P value	S	< 0.0001	< 0.0001	< 0.0001	< 0.0001	< 0.0001
	MS	0.540	0.117	0.042	0.098	0.154
	C	0.006	0.098	0.064	0.034	0.463
	DP	< 0.0001	0.023	< 0.0001	< 0.0001	< 0.0001
	S × MS	0.155	< 0.0001	< 0.0001	0.515	< 0.0001
	S × C	< 0.0001	0.019	< 0.0001	0.022	< 0.0001
	S × DP	0.002	0.027	0.226	0.003	0.262
	MS × C	0.133	0.500	0.813	0.082	0.154
	MS × DP	0.621	0.681	0.265	0.852	0.152
	C × DP	0.942	0.994	0.989	0.888	0.864
	S × MS × C	< 0.0001	0.018	0.231	0.001	0.037
	S × C × DP	0.635	0.874	0.652	0.491	0.734
	MS × C × DP	0.725	0.952	0.755	0.549	0.494
	S × MS × C × DP	0.130	0.301	0.573	0.056	0.316

Different lowercase letters in the same column represent significant difference between experiment seasons, crops, dual-purpose, or maturity stages (*P* < 0.05). *Tr* Transpiration rate, *Pn* Net photosynthetic rate, *Ci* Intercellular carbon dioxide concentration, *Gs* Pore conductivity of water vapor, *WUE* Water use efficiency

### Influence of season and crop on antioxidant properties

As presented in Table 5, the interaction between season and crop had significant (*P* < 0.001) effects on the catalase activity and free amino acid and malondialdehyde contents. The interaction of season and dual-purpose had a significant (*P* < 0.05) effect on the total phenolic content. The interaction of season, crop, and dual-purpose had a significant (*P* < 0.001) effect on the free amino acid content. Season 3 had the highest peroxidase and polyphenol activities but significantly lower (*P* < 0.001) catalase activity and free amino acid, malondialdehyde, water-soluble carbohydrates, and total phenolic contents than seasons 1 and 2. Among the four crops, barley had the highest level of peroxidase activity while oat had the highest levels of catalase and ascorbate peroxidase activities and free amino acid and malondialdehyde contents. Except for peroxidase activity and malondialdehyde content, grain + forage reduced the antioxidant properties of crops (including enzyme activity and chemical properties) compared to grain only.

**Table 5:** Antioxidant properties and yield of different seasons, crops, and dual-purpose (*n* = 66)

Seasons and treatments		Peroxidase (OD470 min^− 1^ mg^− 1^ FM)	Polyphenol oxidase (OD420 min^− 1^ mg^− 1^ FM)	Catalase (OD240 min^− 1^ g^− 1^ FM)	Ascorbate peroxidase (OD290 min^− 1^ g^− 1^ FM)	Free amino acid (mg g^− 1^)	Malonaldehyde (µmol g^− 1^)	Water-soluble carbohydrates (g kg^− 1^)	Total phenolic (g kg^− 1^)
Seasons (S)	Season 1	738 ± 34.1b	10.7 ± 0.72b	38.3 ± 1.21b	127 ± 29.5a	8.27 ± 041a	16.9 ± 1.10a	0.42 ± 0.03b	9.10 ± 0.33a
	Season 2	596 ± 29.3c	7.68 ± 0.50b	52.6 ± 1.88a	45.4 ± 4.48b	5.99 ± 0.43b	14.2 ± 1.02a	0.54 ± 0.03a	7.00 ± 0.65b
	Season 3	861 ± 32.3a	48.3 ± 14.1a	2.23 ± 0.37c	30.5 ± 6.63b	2.12 ± 0.06c	6.07 ± 0.58b	0.21 ± 0.01c	1.49 ± 0.28c
Crops (C)	Wheat	670 ± 30.1b	34.3 ± 12.4	27.7 ± 4.61b	25.2 ± 4.66b	5.34 ± 0.65bc	12.0 ± 1.01bc	0.41 ± 0.04	7.16 ± 1.07
	Oat	624 ± 27.7b	10.3 ± 1.19	47.0 ± 3.03a	129 ± 50.6a	9.15 ± 0.35a	16.7 ± 1.62a	0.44 ± 0.03	7.51 ± 0.55
	Barley	934 ± 24.7a	9.37 ± 1.70	38.0 ± 6.38ab	76.5 ± 21.8ab	5.85 ± 0.77b	14.8 ± 2.02ab	0.37 ± 0.05	5.47 ± 0.76
	Triticale	619 ± 38.0b	22.3 ± 9.32	26.5 ± 4.25b	72.0 ± 13.6ab	3.85 ± 0.41c	9.56 ± 0.89c	0.42 ± 0.05	5.32 ± 1.00
Dual-purpose (DP)	Grain only	717 ± 30.7	21.1 ± 6.24	35.0 ± 3.79	73.2 ± 17.8	5.91 ± 0.50	12.9 ± 1.10	0.45 ± 0.03	7.08 ± 0.65
	Grain + forage	722 ± 33.4	18.7 ± 6.19	32.4 ± 3.69	68.5 ± 16.5	5.62 ± 0.55	13.0 ± 1.12	0.37 ± 0.03	5.45 ± 0.65
P value	S	< 0.0001	< 0.0001	< 0.0001	0.001	< 0.0001	< 0.0001	< 0.0001	< 0.0001
	C	< 0.0001	0.135	0.028	0.038	< 0.0001	0.008	0.738	0.244
	DP	0.909	0.790	0.619	0.848	0.695	0.915	0.093	0.080
	S × C	0.137	0.108	< 0.0001	0.176	< 0.0001	0.001	0.830	0.176
	S × DP	0.605	0.994	0.808	0.979	0.809	0.952	0.038	0.024
	C × DP	0.770	0.356	0.998	0.858	0.840	0.622	0.855	0.961
	S × C × DP	0.344	0.120	0.952	0.986	< 0.0001	0.116	0.617	0.203

Different lowercase letters in the same column represent significant difference between experiment seasons, crops, or dual-purpose (*P* < 0.05)

### Correlation among all variables and yield

**Fig. 2:**
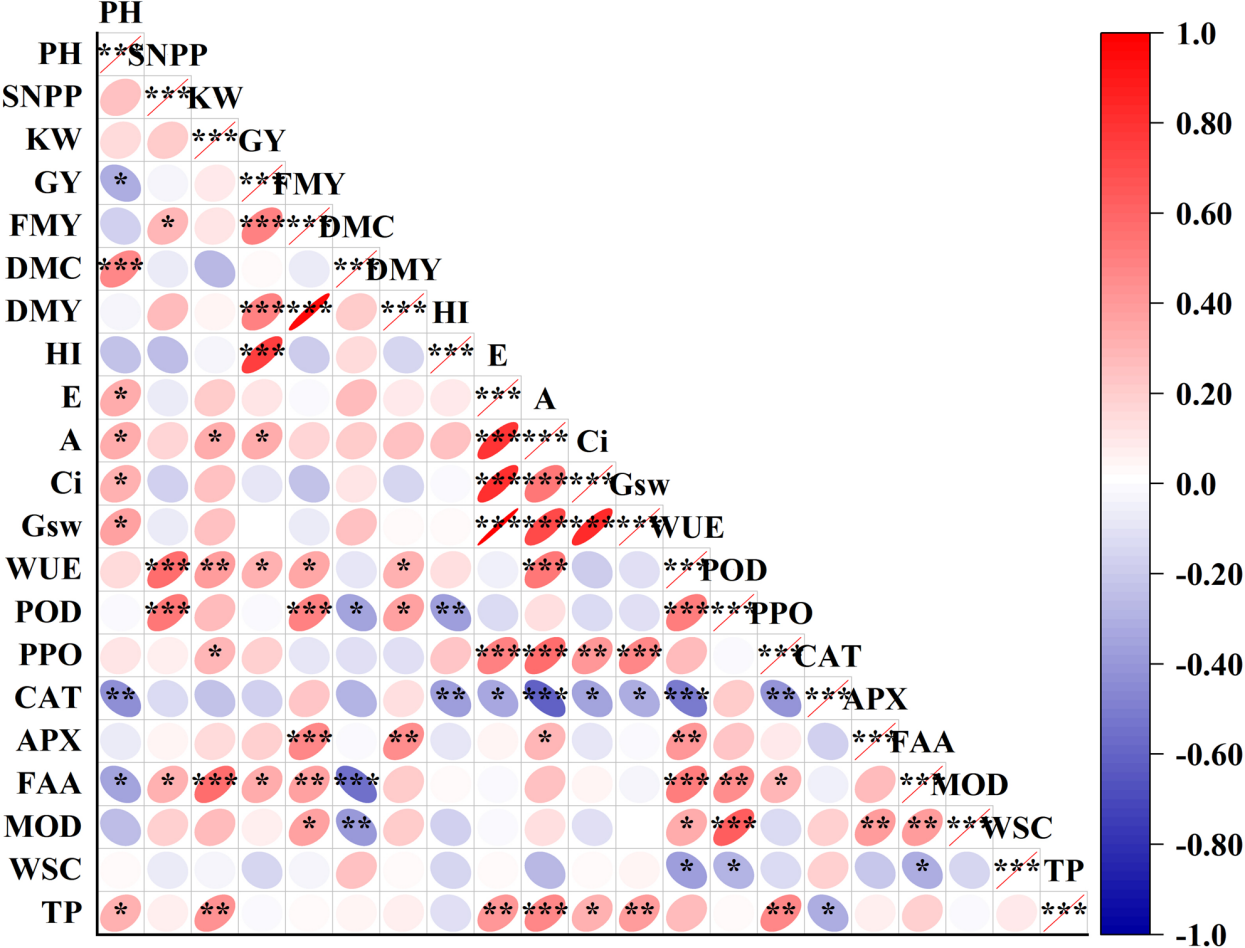
Correlation plot of Pearson among agronomic traits, yield, photosynthetic, and antioxidant properties. Note: Asterisks indicate significant differences at P < 0.05 (*), P < 0.01 (**), and P < 0.001 (***), respectively. PH, plant height; SNPP, stem number per plant; KW, kernel weight; GY, grain yield; FMY, fresh matter yield; DMC, dry matter content; DMY, dry matter yield; HI, harvest index; Tr, transpiration rate; Pn, net photosynthetic rate; Ci, intercellular carbon dioxide concentration; Gs, pore conductivity of water vapor; WUE, water use efficiency; POD, peroxidase; PPO, polyphenol oxidase; CAT, catalase; APX, ascorbate peroxidase; FAA, free amino acid; MOD, malonaldehyde; WSC, water-soluble carbohydrates; TP, total phenolic

To determine the effects of photosynthetic and antioxidant properties on crop agronomic traits and yield, a heat map of the correlations was obtained using the Mantel test (Fig. 2). Overall, the photosynthetic properties (Pn, Tr, Ci, and Gs) had a significant positive effect (*P* < 0.05) on plant height, while catalase activity and free amino acid content had a significant negative effect (*P* < 0.05) on plant height. A significant positive correlation was observed between WUE and stem number per plant, kernel weight, and yield (grain, fresh matter, and dry matter). Among the antioxidant indicators, polyphenol oxidase activity, free amino acid content, and total phenol content were positively correlated (*P* < 0.05) with kernel weight. A positive correlation (*P* < 0.05) was observed between grain yield and free amino acid content (*P* < 0.05).

In terms of the impact of antioxidant properties on yield and harvest index, the free amino acid content had the greatest influence (R^2^ = 0.3148) on kernel weight, followed by the total phenol content (R^2^ = 0.1895) (Figure S1). Similar to the kernel weight, the free amino acid content had the most significant effect (R^2^ = 0.1041) on the grain yield (Figure S2). Although a significant positive (*P* < 0.05) correlation was observed between fresh matter yield and peroxidase and ascorbate peroxidase activities and free amino acid and malondialdehyde content, peroxidase had a more significant effect (R^2^ = 0.1885) on fresh matter yield (Figure S3). Except for polyphenol oxidase activity, almost all antioxidant indicators were negatively correlated with the harvest index (Figure S4). The linear regression model constructed based on the stepwise method also confirmed the above results (Table 6). Overall, the determination coefficients (R^2^) of the antioxidant properties for kernel weight, grain yield, fresh matter yield, and harvest index were different.

**Table 6:** Effect of antioxidant properties on yield

Items	Linear regression equation (stepwise method)
Kernel weight	y = 14.489 + 1.29$\mathrm{x}$_1_+0.764$\mathrm{x}$_2_, $\mathrm{x}$_1_: free amino acid; $\mathrm{x}$_2_: total phenolic
Grain yield	y = 1.457 + 0.177$\mathrm{x}$_1_, $\mathrm{x}$_1_: free amino acid
Fresh matter yield	y = 2.66 + 0.013$\mathrm{x}$_1_-2.44$\mathrm{x}$_2_, $\mathrm{x}$_1_: peroxidase ; $\mathrm{x}$_2_: malonaldehyde
Harvest index	y = 0.731+$\mathrm{x}$_1_-0.004$\mathrm{x}$_2_, $\mathrm{x}$_1_: peroxidase ; $\mathrm{x}$_2_: catalase

The regression models were established using a stepwise selection method to identify potential predictor variables. This analysis is exploratory in nature; therefore, the selected variables represent statistical associations and should not be interpreted as definitive evidence of robust causal relationships

**Fig. 3:**
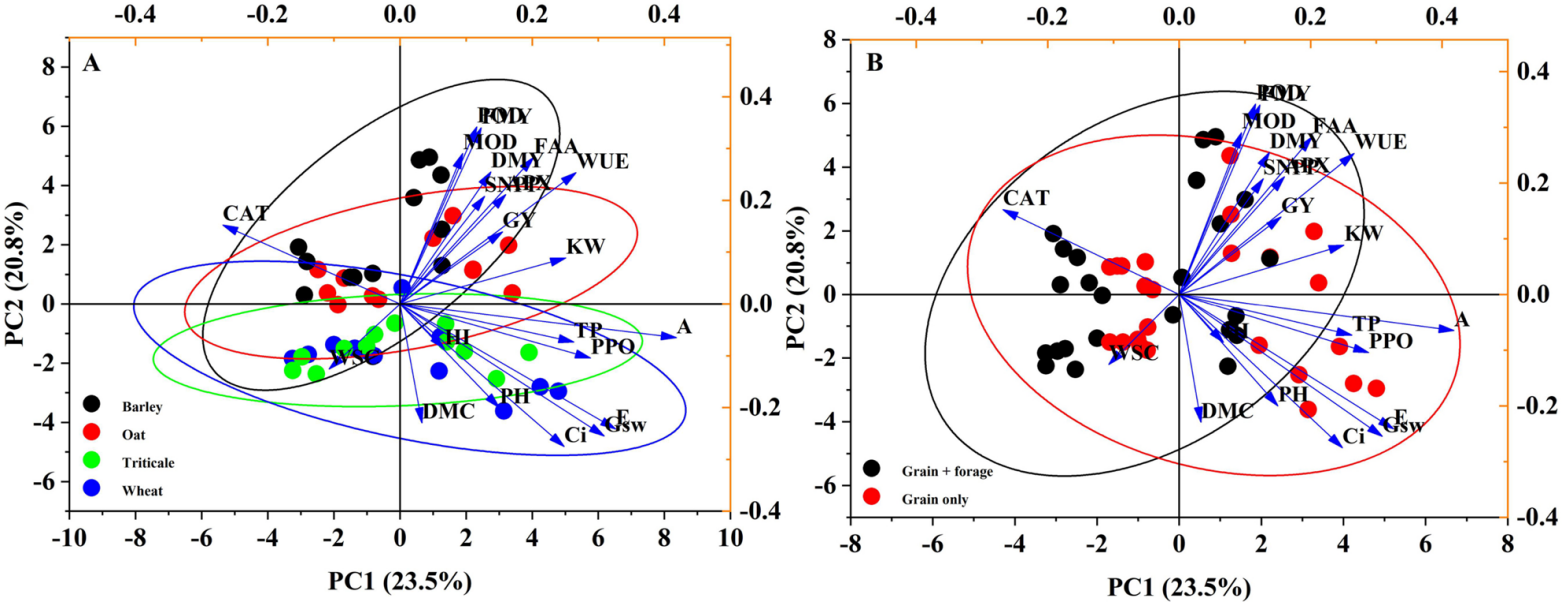
Scatter diagram of principal component analysis of agronomic traits, yield, photosynthetic, and antioxidant properties of different crops (**A**) and dual-purpose (**B**). Note: Ellipses represent a 95% confidence interval. PH, plant height; SNPP, stem number per plant; KW, kernel weight; GY, grain yield; FMY, fresh matter yield; DMC, dry matter content; DMY, dry matter yield; HI, harvest index; Tr, transpiration rate; Pn, net photosynthetic rate; Ci, intercellular carbon dioxide concentration; Gs, pore conductivity of water vapor; WUE, water use efficiency; POD, peroxidase; PPO, polyphenol oxidase; CAT, catalase; APX, ascorbate peroxidase; FAA, free amino acid; MOD, malonaldehyde; WSC, water-soluble carbohydrates; TP, total phenolic

PCA was conducted to visualize the integral relationship among treatments. The first two components explained 44.3% of the total variance. As shown in Fig. 3, a high degree of overlap in the 95% confidence intervals was observed among the four crops (Fig. 3A) and between the two dual-purpose treatments (Fig. 3B). This overlap indicates that the overall physiological coordination among the crops remained relatively stable regardless of the treatment. However, when yield was excluded to focus purely on physiological traits (13 indicators), the factor analysis model revealed a clear hierarchical influence of the experimental factors: season 1 > season 2 > season 3 (season), oat > wheat > triticale > barley (crop), and grain only > grain + forage (dual-purpose) (Table S1).

## Discussion

### Cutting provides more forage resources, but the post-cutting growth rate of wheat was the lowest

In this study, the pre-cutting growth rate in winter ranged from 2.62 to 2.66 t hm^− 2^ FM, which was slightly higher than that of dual-purpose crops in high-rainfall areas of Australia [7] and the Loess Plateau of China [1]. Previous studies have confirmed that the fresh matter yield before cutting is closely related to the crop growth period [6], rainfall [26], and crop variety [5]. However, in our study, the growing season did not significantly affect pre-cutting or post-cutting growth rates. This stability suggests that despite climatic variations (temperature and precipitation) across years, the crops maintained consistent vegetative establishment and environmental adaptability.

Nevertheless, discrepancies in the pre-cutting growth rates likely reflected the sensitivity of the four crops during the early growth stages in different seasons, which is potentially linked to early physiological processes, such as seed germination and stem and leaf establishment. The differences in crop growth stages at the time of cutting also corroborated this phenomenon (Table 1). Notably, wheat exhibited a lower post-cutting growth rate, which demonstrated that cutting reduced the potential yield of wheat as a forage crop (the decrease in yield included both grain and fresh matter yields). In addition, among the four crops, wheat was susceptible to damage from cutting, resulting in damage to tillering and ear fertility and ultimately impacting yield [10]. In contrast, oat and triticale had the highest fresh and dry matter yields in the post-cutting growth stage, respectively, indicating that they were not affected by cutting and had a high forage potential. It is also worth noting that the interaction between crop type and season only influenced the pre-cutting growth rate. This interaction may stem from the complex interplay between crops and their environment based on factors, such as crop adaptability to specific environmental conditions (including light, rainfall, temperature, and harvesting) and differences in physiological regulation mechanisms. Unfortunately, this study did not measure physiological indicators (such as enzyme activity and hormone levels) in the early stages of crop growth and thus, could not provide deeper insights into these mechanisms.

### Seasons, cutting, photosynthesis, and antioxidant properties impacted grain and fresh matter yields and forage potential of crop

Rainfall availability was a primary driver of fresh matter yield. In this study, during growing seasons 2 and 3, lower rainfall led to prolonged drought stress for the crops, inhibiting nitrogen absorption by the root system [27], subsequently reducing the expansion of leaf area and diminishing the ability of leaves to capture resources (i.e., water, CO_2_, solar radiation, and mineral nutrients) necessary for increased biomass and yield [28]. Conversely, growing season 1 experienced sufficient rainfall in the early stages, thus providing ample moisture for crop tillering (Fig. 1), which created conditions conducive to the enhancement of fresh matter yield. Therefore, the photosynthetic properties and WUE during season 1 were significantly higher than those during seasons 2 and 3 (Table 4).

Our multivariate analysis (PCA) provided statistical reinforcement for these environmental observations. The high degree of overlap observed in the PCA (Fig. 3) suggests that the fundamental physiological strategies of these four cereal crops are relatively conserved. However, the Factor Analysis (Table S1) clarified the hierarchy of influence, indicating that seasonal variation (driven primarily by rainfall) outweighed the effects of crop species or dual-purpose management. This implies that under the variable climatic conditions of the Yunnan Plateau, environmental constraints—specifically rainfall stability—are the primary determinants of crop physiology and final yield, rather than the specific agronomic management (cutting) applied. This statistical finding aligns with the observed yield data, where season-to-season yield gaps were more pronounced than differences between management treatments.

The interplay between tillering and yield trade-offs was also evident. The fresh matter yield was the highest in growing season 3 due to its lower maturity compared to that of growing seasons 1 and 2, which likely resulted in a higher water content, thereby increasing the fresh matter yield. As the number of tillers increased, intense competition for resources arose between each tiller and the main stem. This competition can lead to an uneven distribution of nutrients and water between the tillers and main stem, thereby affecting the plumpness and weight of the grains [29]. However, among the four crops, wheat was most susceptible to the combined effect of reduced rainfall and cutting damage, resulting in a decrease in the number of ears and grains. Paradoxically, this also mitigated the nutritional competition between tillers and the main stem, ultimately leading to the lowest grain yield but highest stem number per plant (Table 3). However, numerous studies have clearly indicated that the amount of rainfall is a crucial factor determining the forage and grain yields of these crops [2, 26, 30]. To compensate for potential yield declines (including grain and fresh matter) caused by insufficient rainfall, agricultural practices such as adjusting sowing densities [31], optimizing cutting timing [32], applying nitrogen fertilizers appropriately [33], and irrigation [34] have been employed to effectively mitigate the risk of crop yield reduction.

In this study, the photosynthetic properties at the milk stage were higher than those at the dough stage. This was attributed to the fact that at the milk stage, the leaf area of the crop fully expanded (reaching its maximum extent), which was accompanied by a peak in both the number and function of chloroplasts, leading to a significant enhancement in photosynthetic efficiency. Consequently, this inevitably boosted the transpiration pull, further facilitating the transportation of water and nutrients within the plant body to meet the developmental demands of the grains [35]. Simultaneously, CO_2_ from the air was continually replenished in the leaf cells, thereby enabling the Ci to be maintained at a relatively high level [36]. Additionally, as the crop enters the physiological maturity at the dough stage, leaf functions gradually decline, with a reduction in the activity of photosynthesis-related enzymes, consequently diminishing the photosynthetic capacity of the leaves [37]. Generally, cutting delayed crop maturity [38]. As the growth period extended, the stages among the crops converged (grain only and grain + forage) in this study; however, even at harvest, the maturity of the grain only treatment remained higher than that of the grain + forage treatment (Table 1). During this process, particularly during the crop maturity stage, high temperatures may have forced the crops to mature prematurely, leading to a sharp decline in photosynthetic properties and in inadequate grain filling [39].

Antioxidant activity served as a key physiological indicator of crop response to cutting stress. While cutting reduced the photosynthetic source (leaf area), it triggered a stress response regulated by antioxidant enzymes [40, 41]. In the present study, although there were no differences in peroxidase activity and malondialdehyde content between the grain only and grain + forage treatments (Table 5), these two indicators were the most significant factors affecting fresh matter yield (Table 6). Interestingly, these indicators (peroxidase activity and malondialdehyde content) showed slightly higher values in the grain + forage treatment than those in the grain-only treatment. This also confirms that cutting had a pronounced negative impact on crop growth. This finding was consistent with previous studies [42–44].

Free amino acid content represents one of the major forms of nitrogen storage in plants [45], and a higher free amino acid content can facilitate the transport and distribution of amino acids to the grain, subsequently increasing grain weight [46]. Unlike fresh matter yield, grain yield was primarily regulated by the free amino acid content in this study (Table 6). Increasing the application of nitrogen fertilizers can directly elevate the level of nitrogen within plants, subsequently promoting the synthesis and accumulation of free amino acid. Consequently, some studies have aimed to mitigate the negative impacts of dual-purpose cropping on grain yield by enhancing nitrogen fertilizer application, thereby achieving positive outcomes [47, 48]. In addition to being influenced by the free amino acid content, kernel weight was also affected by the total phenol content. Total phenols are secondary metabolites with antioxidant properties that protect plant cells [49]. During crop growth and development, total phenols may reduce oxidative stress-induced cellular damage and maintain normal cellular functions and metabolic activity [50].

Overall, when considering only the photosynthetic and antioxidant properties of the crops and dual-purpose system among the four crops, only oat and grains received the highest ratings. This indicates that dual-purpose utilization had the least impact on the normal growth of oat. Despite the in-depth exploration of the relationship between photosynthetic or antioxidant properties and crop growth in this study, this research was constrained by the experimental conditions, which failed to comprehensively consider the interactions with other environmental factors, such as soil fertility and pest infestation. Furthermore, the limited sample size might have affected the universality of the conclusions. Future studies should expand the sample range and incorporate multifactorial analyses.

### Evaluation of the economic benefits of dual-purpose crops and prediction of grain and forage yields

To provide a comprehensive evaluation for producers, we theoretically assessed two additional utilization scenarios: forage-only (whole-plant silage) and forage + grain. While grain yield is a primary income determinant [2], the nutritional value of forage is increasingly recognized as a key economic driver [51, 52]. While the current study did not directly analyze the forage value of crops, prior research has already illuminated the significant nutritional advantages of whole-plant cereals [53], particularly after at the milk stage, because of their high starch content [54]. Moreover, a high forage value is maintained at the dough stage [55]. Notably, compared to the singular harvest of grain or forage (whole-plant), the harvest of grain + forage significantly reduced the content of neutral detergent fiber and acid detergent fiber [56], thereby improving forage quality and potential market value.

Economic analysis revealed distinct trade-offs between the systems. The theoretical income models for different utilization strategies (grain-only, forage-only, and grain + forage) are presented in Table 7, providing the analytical framework for this assessment. Although the dual-purpose strategy reduced grain yield, it balanced or even exceeded the total income of the grain-only system by generating additional revenue from the forage harvest. This finding aligns with studies from Australia [9], the United States [57], and China [1, 6] revealed the revenue advantages of the grain + forage. The key to maximizing the benefits of this model lies in rational market price allocation (for grains, high-quality forage, and low-quality forage), which highlights the significance of market conditions in shaping agricultural production decisions [51]. Concurrently, cost control (including costs associated with harvesting grain and fresh matter) is particularly crucial in resource-constrained environments, which require producers to meticulously assess the balance between additional forage revenue and the loss of grain yields.

**Table 7:** Production cost and income of dual-purpose crop

Dual-purpose	Formula	Explanation	Note
Grain + only	y = Ax-B_1_	y, income; x, grain yield; A, grain price; B_1_, cost	(1) B_2_, B_3_, and B_4_ > B_1_; (2) B_4_ and B_2_ > B_3_ and B_1_; (3) x_2_ > x_3_; (4) x> x_4_
Forage + only (cutting + whole-crop)	y=Cx_1_+Dx_2_-B_2_	y, income; x_1_, forage yield of cut; x_2_, whole-crop yield; C, price of high-quality forage (DC24-29); D, price of low-quality forage (DC77-92); B_2_, cost	
Forage + only (whole-crop)	y=Dx_3_-B_3_	y, income; x_3_, whole-crop yield; D, price of low-quality forage; B_3_, cost	
Grain + forage	y=Ax_4_+Cx_1_-B_4_	y, income; x_4_, grain yield; x_1_, forage yield of cut; A, grain price; C, price of high-quality forage (DC24-29); B_4_, cost	

From a sustainability perspective, the dual-purpose system demonstrated superior resource use efficiency. Despite lower absolute grain and biomass yields, the dual-purpose treatment resulted in a higher harvest index (Table 3). This indicates a more efficient conversion of biomass into harvestable products [6, 58]. In summary, while the dual-purpose model requires careful management of the grain-forage trade-off, it offers a resilient strategy to maximize economic returns and resource efficiency, particularly in variable market conditions.

### Dual-purpose crops and the implementation of agronomic management measures were restricted by regional environments, particularly rainfall

The success of dual-purpose systems is fundamentally constrained by regional environments, with rainfall serving as the critical bottleneck. Globally, management strategies have adapted to local precipitation patterns (Tables 2, 3 and 8 and S1). In semi-arid regions like China’s Loess Plateau [6, 31] and Iran’s Karaj area [59], tend to cultivate drought-tolerant crops like wheat and barley. Conversely, in high-rainfall regions like the Himalayan foothills [60] and Brazil’s Paraná Plateau [61], support diverse crop cultivation, including wheat, barley, oats, and rye, vividly demonstrating the decisive impact of rainfall on crop selection.

**Table 8:** Representative countries of dual-purpose crop cultivation and economic benefits

Countries	Representative regions and annual rainfall	Crop type	Agricultural management and economic benefits
China	Loess Plateau (400–600 mm)	Wheat	Delayed sowing (2 Oct.) resulted in a 33.4% reduction in forage yield, but an additional forage was obtained (1.6 t DM hm^-2^), and spring defoliation resulted in a decrease in grain yield [31]. Dual-purpose crop management could increase economic benefits by 15% [6]. Root pruning was beneficial to improve water use efficiency and yield of dual-purpose wheat. Cutting had no effect on yield in dry season, but reduces biomass and grain yield in rainy season [44].
Australia	High-rainfall zones (>600)	Wheat	Delayed cutting of wheat resulted in a 40% reduction in grain yield, but early cutting had no effect on grain yield and quality [64]. Dual-purpose provided 3.1~3.4 t DM hm^-2^ and 2.3~2.4 t DM hm^-2^ of forage, respectively [7]. When dual-purpose crop were used for grazing, stocking rate was significantly positively correlated with animal body weight [62].
India	Himalayas region (700–1000 mm)	Wheat	Dual-purpose wheat provides 2.63-3.77 t·hm^-2^ of fresh grass, but cutting significantly reduces the dry matter accumulation rate of wheat [60].
Egypt	Mediterranean region (300–500 mm)	Wheat, barley, oats, and triticale	Forage resources obtained 60 d after sowing could be replace 2% of commercial forage [63]. Dual-purpose barley management could increase economic benefits by 7.40%~11.4% [64].
Turkey	Konya region (300–500 mm)	Wheat and barley	Dual-purpose barley management could be increase economic benefits by 44.6%~61.1% [65]. Compared to grain + only, dual-purpose management only affects the 1000-grain weight of wheat [66].
Iran	Karaj region (200–400 mm)	Barley	The economic benefits increased by 23.9%~43.6% [59]. Dual-use management led to a decrease in grain yield, but the top dressing of nitrogen fertilizers could minimize this negative impact [67].
America	Texas region (300–600 mm)	Wheat	Forage yield was affected by different field management [68]. Grain yield increased with increasing seeding rate, but forage yield decreased with increasing cutting frequency [69].
Brazil	Paraná Plateau region (1000–1500 mm)	Wheat	Although dairy cows gain weight depending on the number of days grazing [70], dual-purpose wheat could be used in grazing systems in southern Brazil during periods of forage shortage without negatively impacting grain yield [61].

In response to varying rainfall conditions, agricultural management strategies also exhibit notable regional variations. Dry regions focus on the meticulous utilization of water resources and attempt to enhance WUE by methods such as delaying sowing times [71] and implementing root pruning techniques [32]. Conversely, wet regions emphasize optimizing crop growth cycles for maximum yields, flexibly adjusting harvesting strategies to balance forage and grain outputs [8, 72]. In conclusion, the promotion of dual-purpose crop management models, has emerged as an effective means to enhance agricultural economic benefits in multiple countries. Its success has been validated across a broad spectrum of regions, including China, Australia, Egypt, Turkey, and Iran, with notable economic gains ranging from 10% to 60%, as influenced by factors such as crop types, climatic conditions, technological levels, and market demands (Table 8).

It is noteworthy that different management measures yielded varying results under different climatic contexts. For instance, in China’s Loess Plateau [31, 44], delayed sowing sacrifices some forage yield but leads to additional forage harvests. In contrast, delayed harvesting of wheat in Australia directly leads to a significant decrease in grain yield [73]. Furthermore, root pruning techniques significantly improve WUE and overall yields of dual-purpose wheat in dry regions [44], while moderate nitrogen application in Iran’s Karaj area effectively mitigates the potential negative impact of dual-purpose management on grain yields [1, 67].

While striving for high crop yields, research has also revealed a delicate balance between crop yield and forage quality. In India’s Himalayan region, high forage yields of dual-purpose wheat are accompanied by a decrease in dry matter accumulation rates [60]. In Australia, timely harvesting strategies maintain wheat grain yields and quality, while excessive delays lead to significant yield reductions [73]. More importantly, the successful implementation of dual-purpose crop management models not only promotes agricultural economic benefits but also strengthens the complementary relationship between animal husbandry and agriculture. In regions such as Egypt [63, 64], Turkey [56, 65], and Brazil [61, 70], this model not only provides stable forage resources for animal husbandry but also serves as a crucial feed source for livestock, such as dairy cows during forage shortages, embodying a harmonious vision of coexistence among various components within agricultural ecosystems.

Unlike previous studies that focused broadly on regional or temporal variations [42, 43, 74], this study elucidates the intrinsic physiological mechanisms within a variable climate context (Yunnan Province). Although the findings reveal the inherent high variability of such systems, they also offer a more comprehensive perspective on how temperate dual-purpose crops adapt to environmental stresses, particularly rainfall fluctuations [5, 6, 48, 71, 75]. By integrating analysis of photosynthetic efficiency and antioxidant properties, we provide novel insights into how crops physiologically adapt to the combined stresses of cutting and rainfall fluctuation. This physiological perspective fills a critical knowledge gap regarding crop recovery mechanisms [14, 76, 77], offering a scientific basis for optimizing dual-purpose systems in temperate regions facing environmental uncertainty.

## Conclusion

The results of the experimental study revealed that although grain + forage decreases grain and fresh matter yields, this system possesses significant potential to compensate for or even exceed the revenue generated by grain yield through forage income. Furthermore, when evaluating the potential impact of the four forage crops for dual-purposes, this study found that oat was the optimal choice because of its superior performance in balancing yield and profitability. However, when only considering the forage value, barley emerged as a more ideal option. This conclusion underscores the flexibility and economic potential of the dual-purpose for optimizing crop selection based on diverse economic and agricultural criteria. Based on the findings of this study, future research should expand the variety and scope of dual-purpose crops, incorporating multi-year validations across diverse environments (such as varying altitudes, temperatures, and rainfall patterns) to comprehensively enhance the applicability and value of the research.

## Supplementary Information

Supplementary Material 1.

## Data Availability

The raw data supporting the findings of this study are available from the corresponding author upon reasonable request.

## Abbreviations

Pn: Net photosynthetic rate
Ci: Intercellular carbon dioxide concentration
Tr: Transpiration rate
Gs: Pore conductivity of the water vapor
WUE: Water use efficiency
PCA: Principal component analysis
