# Supplementary material for: Balancing grain and forage production in dual-purpose cereals: physiological basis, yield variation, and economic evaluation
Source: BMC Plant Biol. 2026 Feb 26;26:597. doi: 10.1186/s12870-026-08446-5 (PMC13040776; doi:10.1186/s12870-026-08446-5)

Table S1 Scores and rankings of seasons, crops, and dual-purpose (based on photosynthetic and antioxidant properties)

| Seasons and treatments | | Factor analysis value | | | Cumulative variance contribution rate (initial eigenvalue > 1) | Composite scores | Ranking in composite scores |
| --- | --- | --- | --- | --- | --- | --- | --- |
|  |  | FAC 1 | FAC 2 | FAC3 |  |  |  |
| Seasons | Season 1 | 0.616 | 1.023 | -0.0597 | Initial eigenvalue of principal component 1=40.8%, Initial eigenvalue of principal component 2=18.9%, Initial eigenvalue of principal component 3=13.5% | 0.6943 | 1 |
|  | Season 2 | -0.4027 | -0.4438 | 1.047 |  | 0.1520 | 2 |
|  | Season 3 | -0.2843 | -0.7723 | -1.317 |  | -0.1816 | 3 |
| Crops | Wheat | 0.3892 | -0.3766 | 0.0101 |  | 0.3473 | 2 |
|  | Oat | -0.0431 | 0.5824 | 0.488 |  | 0.4165 | 1 |
|  | Barley | -0.5615 | 0.4651 | -0.2971 |  | 0.0765 | 4 |
|  | Triticale | 0.2010 | -0.4768 | -0.0384 |  | 0.2452 | 3 |
| Dual-purpose | Grain only | 0.3715 | -0.0747 | 0.0859 |  | 0.4075 | 1 |
|  | Grain + forage | -0.3715 | 0.0747 | -0.0869 |  | 0.1089 | 2 |

**Figure S1.** Effect of antioxidant properties on kernel weight

Note: Projection represents a 95% confidence interval.


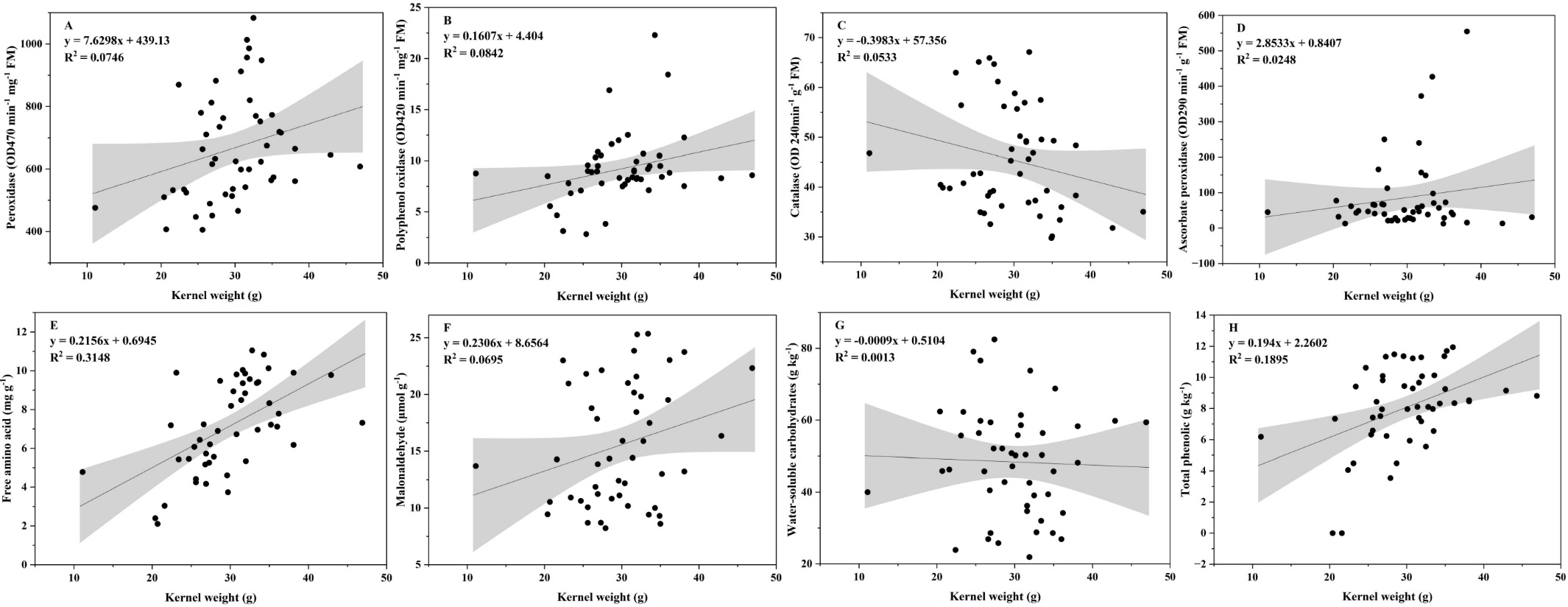


**Figure S2.** Effect of antioxidant properties on grain yield

Note: Projection represents a 95% confidence interval.


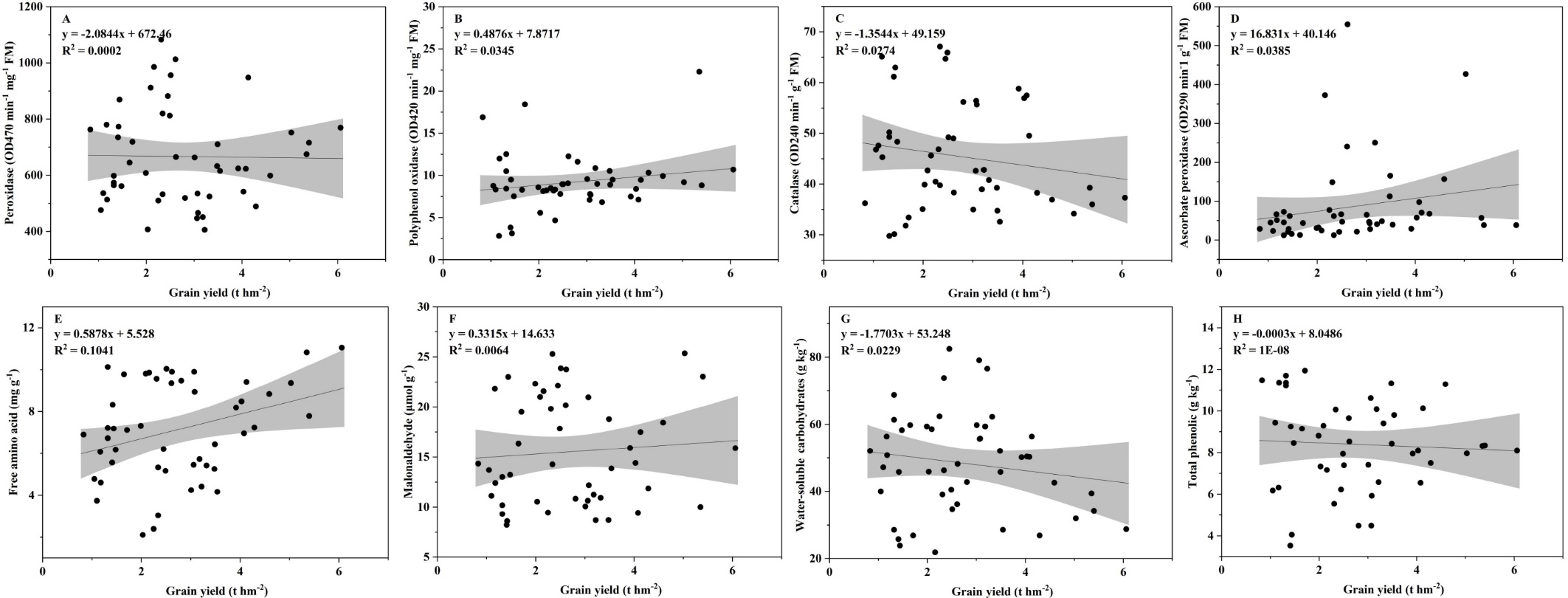


**Figure S3.** Effect of antioxidant properties on fresh matter yield

Note: Projection represents a 95% confidence interval.


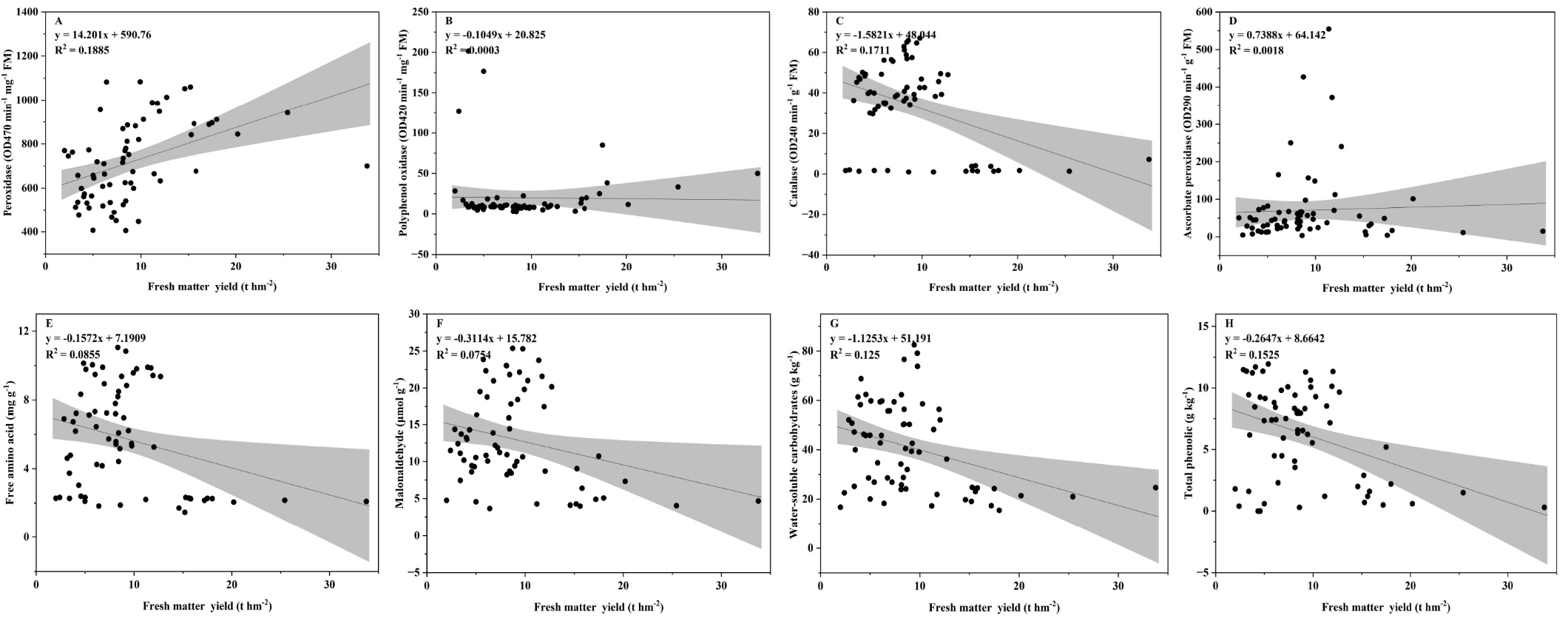


**Figure S4.** Effect of antioxidant properties on harvest index

Note: Projection represents a 95% confidence interval.


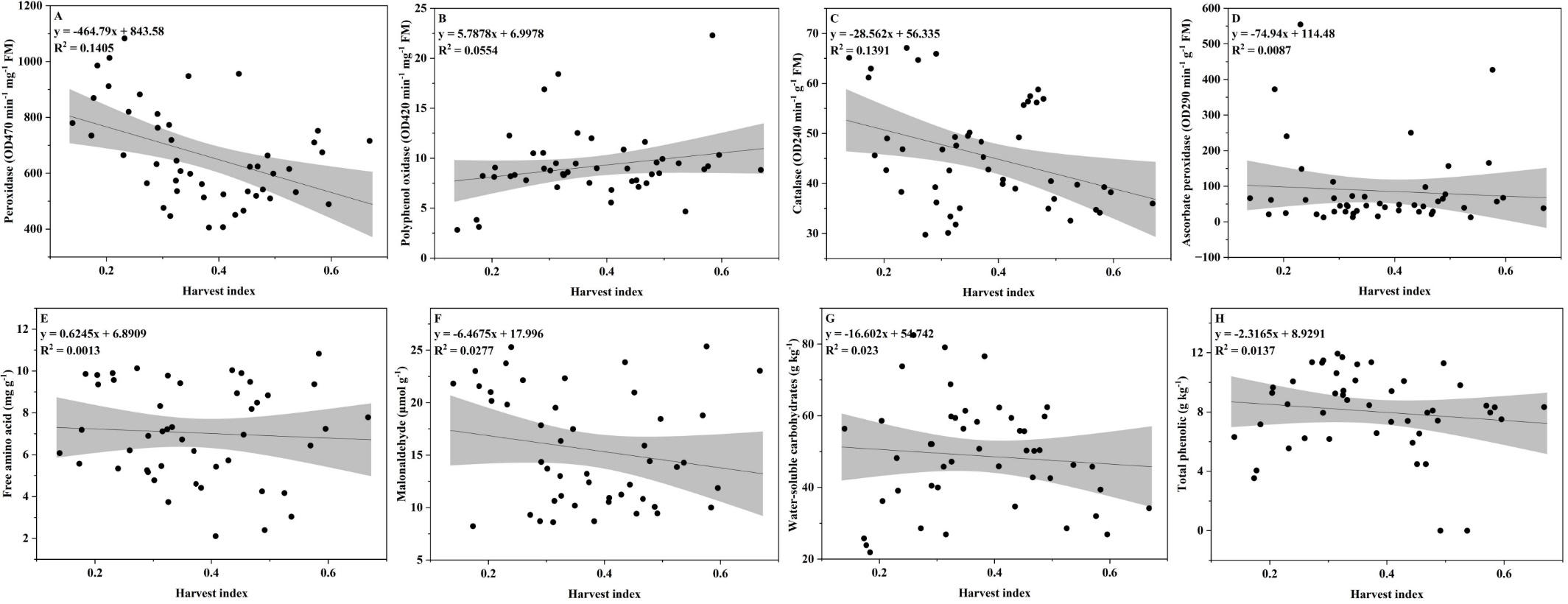

Supplement: Supplementary file 1 — Supplementary Material 1. [file 12870_2026_8446_MOESM1_ESM.docx]
